# The Artificial Third: A Broad View of the Effects of Introducing Generative Artificial Intelligence on Psychotherapy

**DOI:** 10.2196/54781

**Published:** 2024-05-23

**Authors:** Yuval Haber, Inbar Levkovich, Dorit Hadar-Shoval, Zohar Elyoseph

**Affiliations:** 1 The PhD Program of Hermeneutics and Cultural Studies, Interdisciplinary Studies Unit, Bar-Ilan University, Ramat Gan, Israel; 2 Tel-Hai Academic College, Kiryat Shmona, Israel; 3 Department of Psychology and Educational Counseling, The Max Stern Yezreel Valley College, Emek Yezreel, Israel; 4 Department of Brain Sciences, Faculty of Medicine, Imperial College London, London, United Kingdom; 5 The Center for Psychobiological Research, Department of Psychology and Educational Counseling, The Max Stern Yezreel Valley College, Emek Yezreel, Israel

**Keywords:** psychoanalysis, generative artificial intelligence, psychotherapy, large language models, narcissism, narcissist, narcissistic, perception, perceptions, critical thinking, transparency, autonomy, mental health, interpersonal, LLM, LLMs, language model, language models, artificial intelligence, generative, AI, ethic, ethics, ethical

## Abstract

This paper explores a significant shift in the field of mental health in general and psychotherapy in particular following generative artificial intelligence’s new capabilities in processing and generating humanlike language. Following Freud, this lingo-technological development is conceptualized as the “fourth narcissistic blow” that science inflicts on humanity. We argue that this narcissistic blow has a potentially dramatic influence on perceptions of human society, interrelationships, and the self. We should, accordingly, expect dramatic changes in perceptions of the therapeutic act following the emergence of what we term the artificial third in the field of psychotherapy. The introduction of an artificial third marks a critical juncture, prompting us to ask the following important core questions that address two basic elements of critical thinking, namely, transparency and autonomy: (1) What is this new artificial presence in therapy relationships? (2) How does it reshape our perception of ourselves and our interpersonal dynamics? and (3) What remains of the irreplaceable human elements at the core of therapy? Given the ethical implications that arise from these questions, this paper proposes that the artificial third can be a valuable asset when applied with insight and ethical consideration, enhancing but not replacing the human touch in therapy.

## Introduction

### Overview

The introduction of generative artificial intelligence (GAI) has profound implications for a range of human disciplines from education and medicine to economics and law [1-3]. While forms of artificial intelligence (AI) have been around since the 1950s, impacting areas like algorithmic preferences and search functions, it was the launch of large language models (LLMs) in chatbots like ChatGPT in November 2022 that marked a significant milestone [4]. This development catapulted the role of GAI in public discourse to unprecedented prominence, surpassing previous milestones in the field [5].

LLMs are GAI algorithms that harness deep learning and vast data sets to process, summarize, generate, and predict linguistic content [6]. In this paper, we argue that the emergence of GAI is more than just a technical evolution; it is a paradigm shift with deep social and psychological implications [7,8]. It challenges the historic human monopoly over language, with the possibility of shaking the foundation of humanity’s cultural and intellectual exclusivity. In other words, for the first time in history, a nonhuman entity exhibits language processing abilities that in many areas (but still not all) are equal to and sometimes even surpass those of humans. This paper explores the ripple effects of this shift by spotlighting its potential impact on psychotherapy, which is a central domain in mental health care [9-11].

We have chosen to analyze this paradigmatic shift brought about by the development of GAI through the lens of Freud’s concept of “narcissistic blows” to human self-understanding. This psychoanalytic concept aptly describes how major scientific advances, such as the development of GAI, can fundamentally challenge long-held beliefs about human identity and uniqueness. While other theoretical frameworks such as Kuhn’s [12] paradigm shifts could also provide valuable insights into the impact of GAI, we believe that Freud’s theory offers a particularly compelling lens through which to examine the psychological, social, and existential dimensions of this possible technological revolution. The return to Freud’s conceptualization is also valuable because he is considered one of the most influential thinkers in shaping the modern self and is widely regarded as the “father” figure of psychotherapy. Therefore, revisiting his work is particularly relevant when dealing with a transformative change like the one brought about by the introduction of GAI into the psychotherapeutic space. Following this psychoanalytic analysis, we will propose in this paper to conceptualize the entry of GAI into the psychotherapeutic space as an artificial third.

### GAI: The Fourth Narcissistic Blow to Humanity

In 1917, in his article “A Difficulty in the Path of Psycho-Analysis,” Freud [13] assessed how scientific discoveries reshaped our cultural understanding and self-perception. He identified three narcissistic blows inflicted on humanity by science that pushed us to confront and abandon long-held naive beliefs of our narcissistic centrality and control over the world. These paradigm shifts, although jarring, catalyzed tremendous societal advancement [13]. First, the “cosmological” blow came from Copernicus who taught us that the earth orbits the sun and not the other way around [14,15]. Second, Darwin’s “biological” revelation presented humans as just another evolutionary link and not a divine culmination [14,15]. Third, Freud himself introduced the “psychological” blow with his psychoanalytic theory suggesting that beneath our perceived rationality lays unconscious drives and conflicts that are beyond the control of our “ego” [13,14]; by asserting that one is not the master of one’s own house, he implied our limited control over our inner selves [13].

Building on Freud’s framework, modern computer science appears to be delivering a fourth potent blow to human narcissism, which we suggest conceptualizing as the “linguistic narcissistic blow.” This blow is historically profound: after understanding that the earth is not the center of the universe (the “cosmological” blow), recognizing our nonexceptionalism in nature (the “biological” blow), and confronting the turbulent undercurrents of our psyche (the “psychological” blow), we are now also faced with the prospect of sharing our linguistic domain. What was once an exclusive human domain might now be shared with ever more sophisticated artificial entities [15]. It is difficult to exaggerate the drama of the possible loss of human monopoly on language since the natural and primary characteristic of humanity was the ability to control and play with language, namely, to produce signs and symbols that indicate things as well as to ever alter the meanings of these symbols. This is how humans acquired the ability to create a subjective mental image and meaning of the external world. These signs can be not only literal but also symbolic in the form of paintings, symbols, rituals, and more. In other words, language and the ability to play with it are, as we learned from Winnicott [16], the foundation of all human culture.

It should be clarified that we do not claim GAI systems possess human language understanding and control. While GAI systems have demonstrated impressive language processing and generation capabilities, they still do not possess genuine understanding or comprehension of language in the same way that humans do [17]. These systems operate based on complex statistical patterns and associations learned from vast amounts of training data, but they lack the rich contextual knowledge, reasoning abilities, and embodied experience that underpin human language use [18]. Nonetheless, the ability of GAI systems to generate highly coherent and contextually appropriate linguistic outputs has significant and dramatic implications for a range of domains, including psychotherapy.

As we stand on this precipice where GAI entities have an increasing ability to process and produce language, we face a new era brimming with both potential and intricate challenges, particularly in the sphere of mental health [8-10,17].

### Integrating GAI Into Psychotherapy

For over a century, psychotherapy, which is a linchpin in mental health, has hinged primarily on the dialogue between therapist and patient. Its origins can be traced back to Freud and Breuer’s [19] seminal work *Studies in Hysteria*, which conceptualized psychotherapy as the “talking cure.” Although myriad forms of psychotherapy have emerged, the central emphasis on the main method of the patient-therapist dialogue remained consistent [20]. In fact, despite extensive technological and theoretical development in the 20th century, the incorporation of technology into the therapeutic field has been surprisingly minimal [20]. Even as advancements like biofeedback, neurofeedback, and virtual reality have arisen, their widespread adoption in therapeutic practice is still limited. However, it seems that GAI, having demonstrated increasingly sophisticated language processing and generation capabilities, stands poised to radically revolutionize the field of psychotherapy.

Wittgenstein’s [21] linguistic theory provides a comprehensive philosophical lens to examine the linguistic capabilities and limitations of GAI in the context of psychotherapy. Wittgenstein’s [21] early work assertion that “we make to ourselves pictures of facts” refers to the way language mediates one’s world into a subjective picture consisting of words. This lingo-philosophical perception encapsulates the potential of GAI to also build a picture of the world from words or, using therapeutic concepts, generate outputs that resemble interpretations, insights, narratives, reflections, validations, and more. Until recently, all of these talk-based psychotherapy capabilities were restricted to mental health professionals; now, thanks to its proficiency in language, GAI is not just an adjunct but may actually redefine the therapeutic landscape. Preliminary research underscores GAI’s prowess in tasks like treatment summarization, risk assessment, and real-time diagnosis, all of which rely on highly strong language processing capabilities [8-10,22].

While Wittgenstein’s [23] early work presents language as a picture of reality, in his later philosophy, he developed the concept of “language-games” [24]. This concept highlights the rule-governed, socially embedded nature of linguistic meaning. Following Wittgenstein’s later work, other linguistic theories such as “speech act” theory [25] also emphasized the social and practical dimensions of language. From this perspective, GAI’s ability to produce meaningful interpretations and to hold the full linguistic understanding necessary for functioning in the therapeutic realm is constrained. This limitation arises from its lack of grounding in the social and normative aspects of language use.

Nevertheless, we argue that in an age when GAI’s linguistic competencies rival or even eclipse those of humans in some (although, as we emphasized, certainly not all) linguistic areas, it is hard to imagine that psychotherapy will remain an untouched bastion, preserving its “No Entry for Technology” doctrine. GAI’s potential integration into mental health and psychotherapy, in particular, heralds both vast opportunities and challenges, with the prospect of reshaping the therapeutic practice [26].

Hence, it is our conviction that mental health professionals bear an ethical responsibility to proactively engage and influence the integration of GAI within the clinical domain. Specifically, the development and application of GAI tools, methodologies, and conceptualizations for psychotherapy in particular, and mental health in general, must involve close collaboration between mental health professionals, AI researchers, and ethicists to ensure alignment with the core values and goals of the profession [7].

To illustrate the potential integration of GAI into the psychotherapeutic process, let us consider a scenario where a GAI system actively participates in a live therapy session. The GAI listens to the dialogue, analyzes the language and sentiment in real time, and provides insights to both the patient and the therapist. For instance, it might highlight patterns in the patient’s narrative that suggest underlying mental risks, cognitive distortions, or emotional conflicts. It could also offer the therapist suggestions for therapeutic interventions based on the patient’s unique profile. After the session, the GAI could provide a summary, identifying key themes and tracking progress over time. Of course, such an application would need to be developed and deployed with utmost care for patient privacy, data security, and both clinical and ethical considerations [27].

GAI can also serve as an innovative playful space that allows for exploring and processing intrapsychic and interpersonal dynamics in new creative ways. To further illustrate this, let us consider another example where the GAI presence within the therapeutic process supports and enhances the technique of externalizing internal psychological pains or conflicts. By using problem externalization from narrative therapy, GAI systems can generate tangible representations of the patient’s inner struggles, either by transforming the internal voice into a visual representation using an image generator or by having the GAI embody the inner voice, allowing the patient to converse with it in the presence of the therapist and explore it together. This innovative approach may potentially assist patients in gaining new perspectives on their internal conflicts, with the therapist guiding the therapeutic process.

These GAI’s abilities to process and analyze the therapeutic dialogue in real-time, offer insights and suggestions, provide postsession summaries, and engage in live dialogue or role-play with a patient and therapist introduce a novel presence that actively reshapes the dynamics and outcomes of the therapeutic process.

### Exploring the Role of GAI in the Therapeutic Triad

The introduction of GAI into psychotherapy raises central questions about its clinical, ethical, and interpersonal implications [18,26]. This paper suggests that to truly grasp the impact of GAI in psychotherapy, one must first recognize the potential dynamics of introducing a third element into the traditional therapist-patient dyad. This dyadic structure, a cornerstone of psychotherapy, has remained largely unchanged over the past century [28]. Yet, it is an oversimplification to view psychotherapy as solely a 2-person interaction, since it has always operated within a more complex triadic framework [29]. In fact, the “third” or “thirdness” as “other” to the dyad holds a paradoxical attitude: the “third” not only threatens the connection of the dyad but also enables it; in other words, it enables the psychotherapeutic act itself.

Due to a lack of scope, this paper can only touch key landmarks in the triadic concept’s genealogy within psychoanalysis [29]. Freud was the first to place the model of triads at the center of psychological and clinical thinking. For him, the ultimate other was represented by the father within the oedipal developmental drama, that is, in the well-known drama of the triangle relationship formed between the child, their mother, and their father in the child’s early years. Subsequent psychoanalytic theories developed this “third” or “thirdness” concept further [13]. For example, Winnicott [16] visualized it as a “transitional space”—a nexus between imagination and reality and the space where creativity and culture can develop. Ogden [30] introduced the important concept of “analytic third,” addressing the “third” as an abstract entity born from the therapist-patient interrelationship. Building on Ogden’s concept, Bar Nes [31] suggested that the emergence of the “analytic third” is facilitated by a combination of verbal and nonverbal communication, which creates a psychic overlap between therapist and patient. Finally, Lacan [32], expanding on Freud’s ideas, posited that the “third” symbolizes the overarching structure of language that assimilates into our unconscious, shaping the individual’s identity and interactions with the world.

In short, the concept of the “third” in the sphere of psychotherapy is not new at all. Nonetheless, its presence is quite complex; it, on the one hand, is ever present in the therapeutic dyad and, on the other hand, always remains the distinct “other.”

### Redefining the Therapeutic Triangle in the Digital Era

As GAI is being embedded in the mental health arena, we are witnessing the emergence of a distinctive triangular dynamic: therapist, patient, and the GAI “third” entity [26]. This GAI “third,” as a continuation of the “third” in the psychoanalytic perspective, remains as the “other” joining the dyad, but the new quality it brings also fundamentally changes the balance of the therapeutic relationship. For the first time, this “third” element becomes tangible, transitioning from a mere conceptual presence to an interactive entity that both therapist and patient can have a dialogue with and not just about. This marks a new horizon in both the triad therapeutic dynamics and the patient-therapist dynamics [33].

Based on psychoanalysis’ evolution of the “third” mentioned above, we propose conceptualizing the presence of GAI in psychotherapy (and mental health in general) as an artificial third. The artificial third refers to the concrete and symbolic expression of GAI in the cultural-political-digital space and its shaping presence in the therapeutic encounter, society, and the self. While the artificial third can appear in therapy as a “transitional space,” thus creating a playful space in accordance with Winnicott’s [16] perception, its influence does not end there: it also shapes our perception of ourselves and the world. To clarify this point, if until the digital age the way to explore our consciousness was through other human consciousnesses, now it seems, the state of affairs is different. For the first time, we are beginning to know ourselves not only through reflections from other humans but also through direct and indirect reflections from AI entities and their control of the digital sphere.

Although human therapists are far from obsolete, the integration of the artificial third might substantially transform therapeutic methodologies, redefining the essence of therapy and the perceptions and roles of both therapists and patients [18]. However, the impact of the artificial third extends beyond merely shaping the therapeutic landscape; it is also intricately crafted by the human experiences and notions that constitute its core training data. This two-way interaction has significant implications for the development and impact of the artificial third in psychotherapy, suggesting that GAI technology is not only a shaping force but is also influenced by the cultural values, biases, and limitations of its human creators and users [34]. With the incorporation of GAI, the field of mental health stands on the cusp of revolutionary advancements, with the promise of enhanced professional opportunities but also the potential for great dangers and pitfalls [35].

### Advantages of Integrating GAI in Psychological Care

The integration of GAI into psychological care augurs transformative new methodologies. Central to these advancements is the potential for vastly improved accessibility to mental health services. Worldwide, numerous barriers, ranging from socioeconomic constraints and geographical distances to linguistic challenges and intricate cultural contexts, limit access to quality care [36,37]. However, the artificial third may stand to bridge these divides, widening the scope of individuals who are able to receive psychological support.

Beyond accessibility, this novel approach may enable a new level of personalization in mental health care. It envisions a therapeutic experience tailored meticulously to each individual and accounting for clinical, linguistic, cultural, and personal characteristics, and promises a significant shift in therapeutic dynamics. Historically, therapeutic expertise has been monopolized by professionals, but the artificial third might, although not necessarily, foster a more collaborative patient-therapist relationship [7,38]. Such a change may place patients at the forefront of their healing journey, empowering them to engage actively in their therapy.

Moreover, the artificial third opens up new pluralistic possibilities, allowing for the integration of established evidence-based approaches like cognitive behavioral treatment and psychodynamic therapy with cutting-edge and emerging psychological frameworks [39,40]. It may also, as mentioned above, create a new playful space that strengthens the relationship between patient and therapist. While still a burgeoning concept, the artificial third holds undeniable promise for redefining psychological care and harmoniously blending accessibility, personalization, and innovation.

### Challenges of Integrating GAI in Psychological Care

The integration of the artificial third into psychological services presents a multifaceted set of challenges. One major concern is the consolidation of vast amounts of patients’ personal data under a handful of dominant corporations. Such centralization could divert the focus from patient-centered care to commercial interests, thus risking service quality [41]. This commercial shift also raises alarms about unauthorized data use or deep analysis of user behaviors without explicit consent, jeopardizing both user confidentiality and overarching ethical standards [36]. Adding to the uncertainty is the often opaque nature of algorithms, which could obscure decision-making processes.

A subtler yet significant concern is the exaggerated reliance on the epistemic authority attributed to the artificial third. As the artificial third becomes more integral, there is a danger that traditional expert voices, like experienced psychologists, could be overshadowed, which could undervalue the importance of human expertise, experience, thought, and insights in therapy. Furthermore, the potential overreliance on GAI tools in therapy could risk diminishing the significance of authentic human connection and empathy in the therapeutic process.

This concern becomes more acute when these tools are developed by mental health professionals themselves. Although these tools can provide distinct advantages, they are not exempt from the ethical dilemmas and possible conflicts of interest that emerge when therapists participate in the development of a GAI product. The substantial risk is that the artificial third may, detrimentally, shift to become the central focus of therapy in such instances, rather than acting as an instrumental aid designed to support the patients’ therapeutic journey. Additionally, with the artificial third still in its infancy, there is a tangible risk of off the mark guidance, which could lead individuals away from tested psychological practices [42]. Finally, while LLMs are perceived as objective and neutral, they were actually put through a training process that aligned their reactions to a specific “value-like” system that is not transparent to the public [34].

The focus of these concerns lies in the lack of a unified regulatory framework for GAI. Although traditional psychological practices are governed by established regulations, GAI systems currently operate without such oversight. This regulatory gap may allow companies to craft their guidelines, potentially leading to variances in standards and a deviation from the trusted norms of mental health care [38,43]. Moreover, preliminary findings suggest that GAI data sets may sometimes produce social, economic, and cultural biases [7] that have the potential to be inadvertently amplified, thus undermining the goal of providing unbiased psychological support. In conclusion, despite its innovative promise, the introduction of GAI in psychotherapy carries inherent risks that may undermine the therapeutic relationship, the therapist’s professionalism, and the ability to promote the patient’s well-being.

## Engaging With the Artificial Third: Three Fundamental Questions

### Overview

The trajectory of mental health and, specifically, the psychotherapeutic realm in the era of the artificial third remains uncertain. However, it is unequivocally clear that the landscape will undergo profound transformations [7-10,22]. In light of this change and uncertainty, it is imperative to arm both therapists and patients with three key questions regarding (1) the nature of the artificial third, (2) our relationship with it, and (3) the role of humanity in the artificial third era. These questions aim to foster a deeper understanding of the artificial third, encourage reflection on our interaction with it, and prompt a consideration of the unique value and position of human beings in this new landscape. While these questions relate also to the broader interface between humans and AI, they hold special relevance for psychotherapy and the larger mental health domain [7,17,22].

These three questions are meticulously designed to advance the principles of transparency and autonomy, which are essential for fostering critical thinking, especially in the context of interacting with AI systems [44,45]. Critical thinking is one of the most important capacities for promoting human freedom and agency [46,47]. By emphasizing transparency—the ability to understand how GAI systems work and what influences their outputs—and autonomy—the ability to maintain independent thought, creation, and decision-making in the face of GAI influence—we aim to highlight the importance of preserving space for critical reflection and self-determination in the era of AI.

### The Nature of the Artificial Third

In opposition to the widespread but simplistic view that regards GAI systems as impartial and objective, we contend they are based on certain values and cultures that are shaped by the critical factor of the alignment process [34]. Understanding the influence of the alignment process is essential for the responsible integration of GAI into psychotherapy. Therapists have a crucial role in the era of the artificial third—to explore and comprehend the alignment mechanisms of the GAI systems they use. This entails recognizing the inherent values, motivations, and limitations of these systems. Therefore, understanding the nature of the artificial third is not merely about operational knowledge but also carries ethical weight, emphasizing the necessity for a deep awareness of this emerging presence within the therapeutic setting. Therapists have a responsibility to share their insights about GAI systems with their patients, incorporating this transparency into the informed consent process, thus ensuring the maintenance of ethical standards in this new therapeutic landscape.

Consequently, we propose the following question as a guiding principle for therapists and patients when considering the use of GAI in psychotherapy: “To what extent do we understand the alignment process and limitations of the GAI system we are working with?” This question prompts us to consider the following subquestions: What are the underlying values, interests, and driving forces? How are responses generated? and Who bears the responsibility for them? Addressing these questions effectively at a societal level and shaping appropriate policies necessitates the implementation of a structured regulatory framework to ensure the responsible and ethical application of AI in the field of mental health.

### Our Relationship With the Artificial Third

As we elaborated on in the paper, the introduction of the artificial third in psychotherapy creates a new therapeutic triangle of therapist, patient, and GAI. Optimally, this entry can promote a playful space in therapy and patients’ well-being; however, negatively, it may lead to a detriment in focusing on the patient’s needs and the therapist’s self-thinking. Therefore, the most important concrete question when examining the effect of the artificial third entry into psychotherapy is to consider “To what extent does the artificial third become central in the therapeutic space, and does this centrality come at the expense of the patient?” This question examines the dynamics of our interaction with this artificial entity and its position in the new therapeutic triangle.

From a more philosophical point of view, we can also ask “how our discourse with the artificial third is shaping our perceptions of the self, the therapeutic act, and the roles of both the patient and therapist?” This reflection, which necessitates further philosophical, clinical, and ethical research, extends far beyond the confines of the therapy room, delving into broader questions of identity and the essence of human relationships and communication in an increasingly digital landscape.

### The Role of Humanity

Rooted in the principle of autonomy [44], this question critically assesses humanity’s latitude with AI and asks “What distinguishes the human subject from the artificial object?” In the realm of mental health, it differentiates between the roles in which AI can be beneficial and those in which it might be detrimental, suggesting that the latter are better left to humans.

Encountering the artificial third as a nonhuman entity presents a unique opportunity: it allows us to reexplore and, perhaps, redefine our humanity. With the presence of this artificial entity in therapeutic sessions, the question of human uniqueness becomes significant. The concrete question that arises from this critical thinking is “What is the added value of the human therapist within the therapeutic space?” As elaborated on in this paper, it seems that while GAI can provide textual analyses at the highest level, the ability for true human experience, thinking, and therapeutic interpretation is still irreplaceable. In this sense, there is a possibility that the encounter with the artificial third, as an entity fundamentally different from humanity, will not harm the human place in the therapeutic process but rather emphasize its uniqueness and special contribution.

## Conclusion

Since the release of GAI systems, numerous studies have been conducted regarding their applications in the field of mental health [2,7-10,22,34,48-53]. However, the current research seeks to examine the entry of this technology from a broader perspective, particularly focusing on its potential impact on psychotherapy.

The entry of GAI into our lives seems to mark a new age characterized by the possible loss of humanity’s historic monopoly over language, conceptualized in this paper as the “fourth narcissistic blow” (the “linguistic narcissistic blow”) inflicted on humans by science. The fourth narcissistic blow refers to the fact that, due to recent scientific and technological progress, artificial entities are starting to display language abilities similar to humans. Yet, this paper also emphasized that although GAI has impressive analytical and processing capabilities, it still lacks some essential social and pragmatic aspects inherent to human language.

Psychotherapy may experience an upheaval following the recent increasing use of GAI, whose presence was defined here as the artificial third. While GAI’s linguistic abilities may challenge traditional psychotherapeutic paradigms, it also unveils opportunities for enriched therapeutic processes, provided these technologies are used with discernment and a deep commitment to the ethical imperatives of therapy, as well as taking into consideration GAI’s limitations and biases. Therefore, we argue that the integration of the artificial third into the therapeutic sphere does not inherently improve or detract from the psychotherapeutic act. Rather, its influence is contingent, relying on the context, the goal, and the way in which it is implemented.

Moreover, the artificial third is neither an unequivocal solution nor a replacement for the quintessentially human aspects of therapy. Instead, it represents a nuanced technological tool that, if integrated carefully, could hold the promise of enhancing therapeutic practice. As we navigate this delicate balance, the imperative remains to safeguard the irreplaceable human connection that lies at the heart of the therapeutic act, ensuring that technology augments rather than diminishes it.

Indeed, in the artificial third era, we need to accept its growing presence while guarding against total dependence. Upholding patient and therapist autonomy together with GAI transparency will be vital for enabling critical thinking, which is important, especially in the fragile realm of mental health. This will allow us to both judiciously leverage GAI’s potential and protect the humanistic essence of therapeutic practice. This exploration of the artificial third in psychotherapy underscores a pivotal juncture in our understanding and practice of therapy.

## Abbreviations

AI: artificial intelligence
GAI: generative artificial intelligence
LLM: large language model
